# Immunomodulation with minocycline rescues retinal degeneration in juvenile neuronal ceroid lipofuscinosis mice highly susceptible to light damage

**DOI:** 10.1242/dmm.033597

**Published:** 2018-09-05

**Authors:** Katharina Dannhausen, Christoph Möhle, Thomas Langmann

**Affiliations:** 1 Laboratory for Experimental Immunology of the Eye, Department of Ophthalmology, University of Cologne, 50931 Cologne, Germany; 2 Center of Excellence for Fluorescent Bioanalytics, University of Regensburg, 93053 Regensburg, Germany; 3 Center for Molecular Medicine Cologne (CMMC), University of Cologne, 50931 Cologne, Germany

**Keywords:** Juvenile neuronal ceroid lipofuscinosis, CLN3, Microglia, Minocycline, Retinal degeneration

## Abstract

Juvenile neuronal ceroid lipofuscinosis (jNCL) is a rare but fatal inherited lysosomal storage disorder mainly affecting children. The disease is caused by mutations in the *CLN3* gene that lead to the accumulation of storage material in many tissues, prominent immune responses and neuronal degeneration. One of the first symptoms is vision loss followed by motor dysfunction and mental decline. The established *Cln3^Δex7/8^* mouse model mimics many pathological features of the human disease except the retinal phenotype, which is very mild and occurs only very late in these mice. Here, we first carefully analyzed the retinal structure and microglia responses in these animals. While prominent autofluorescent spots were present in the fundus, only a moderate reduction of retinal thickness and no prominent microgliosis was seen in young CLN3-deficient mice. We next genetically introduced a light-sensitive RPE65 variant and established a light-damage paradigm that showed a high susceptibility of young *Cln3^Δex7/8^* mice after exposure to 10,000 lux bright light for 30 min. Under these ‘low light’ conditions, CLN3-deficient mice showed a strong retinal degeneration, microglial activation, deposition of autofluorescent material and transcriptomic changes compared to wild-type animals. Finally, we treated the light-exposed *Cln3^Δex7/8^* animals with the immunomodulatory compound minocycline, and thereby rescued the retinal phenotype and diminished microgliosis. Our findings indicate that exposure to specific light conditions accelerates CLN3-dependent retinal degeneration, and that immunomodulation by minocycline could be a possible treatment option to delay vision loss in jNCL patients.

This article has an associated First Person interview with the first author of the paper.

## Abstract (precis)

**Summary:** Here, we established a light-damage paradigm to model retinal degeneration in the juvenile neuronal ceroid lipofuscinosis mouse and showed the beneficial effects of minocycline on retinal pathology.

## INTRODUCTION

Neuronal ceroid lipofuscinoses (NCLs) are a group of rare autosomal recessive lysosomal storage diseases mainly affecting children, with incidence rates between 1.3 and 7 per 100,000 live births (Mole et al., 2011). The most common form is the juvenile subtype (jNCL), affecting children between 5 and 10 years of age. The first symptom is vision loss, followed by mental deterioration, seizures, immune system abnormalities, personality changes and loss of motor abilities resulting in early death in the twenties (Birch, 1999; Castaneda and Pearce, 2008; Schulz et al., 2013; Staropoli et al., 2012). The underlying genetic defects are characterized by mutations in the *CLN3* gene, which encodes the CLN3 protein (also called battenin), a broadly expressed lysosomal transmembrane protein. The most frequent mutation, occurring in over 80% of patients, is the deletion of exons 7 and 8, resulting in a truncated dysfunctional protein (Cotman et al., 2002; Mole and Cotman, 2015).

Several studies have implicated a role for CLN3 in autophagy, endocytosis, synapse formation, intracellular trafficking, calcium homeostasis and regulation of lysosomal pH (An Haack et al., 2011; Cao et al., 2006; Golabek et al., 2000; Lojewski et al., 2014; Luiro et al., 2004, 2001; Uusi-Rauva et al., 2012). Malfunction of the CLN3 protein leads to accumulation of lysosomal storage material, with accumulations mainly composed of subunit c of the mitochondrial ATP synthase (SubC), lipoproteins and glycoproteins (Palmer et al., 1992; Staropoli et al., 2012).

A typical hallmark of neuronal degeneration is microglial reactivity. Microglia are the resident cells of the innate immune system in the central nervous system (CNS). In the healthy retina, they are located in the plexiform layers, and have a typical ramified morphology with small somata and long, branched protrusions (Hume et al., 1983). They continuously scan their environment for changes in neuronal homeostasis (Nimmerjahn et al., 2005). Dying cells can be recognized by a variety of receptors and ligands, leading to a morphological change of microglial cells to an amoeboid shape. Thereby, microglia migrate towards the area of damage and phagocytose debris. Depending on the nature and duration of the stimulus, a chronic activation can occur. As a result, microglial cells show an increased expression of pro-inflammatory mediators and phagocytose healthy photoreceptor cells, leading to damage of the surrounding neuronal tissue (Aslanidis et al., 2015; Kettenmann et al., 2011; Zhao et al., 2015). Microglia activation is often detected before the first signs of neuronal cell death, leading to the hypothesis that early microglial activation may promote the degenerative disease progression (Karlstetter et al., 2010; Langmann, 2007; Pontikis et al., 2004; Schuetz and Thanos, 2004).

Only a few reports have studied the role of glial cells in jNCL pathogenesis. CLN3 deficiency leads to a functional impairment of microglia and a higher sensitivity of neuronal cells to neurotoxic substances *in vitro* (Parviainen et al., 2017; Xiong and Kielian, 2013). Microglia colocalization with neurons expressing high amounts of an antioxidant enzymes was shown in a *Cln3^−/−^* mouse model (Benedict et al., 2007). Additionally, accumulation of autofluorescent material and an upregulation of glial fibrillary acid protein (GFAP) was seen in human retinal cross-sections with CLN3 deficiency (Bensaoula et al., 2000). These findings led to the hypothesis that microglial activation is connected to neuronal cell damage via oxidative stress (Benedict et al., 2007). Furthermore, microglial activation, storage of autofluorescent material, neuronal death and retinal thinning was observed in the jNCL mouse model at 17 months (Cotman et al., 2002; Pontikis et al., 2004; Seigel et al., 2002). Recently, it was shown that treatment with an autophagy enhancer reduced gliosis in old CLN3-deficient mice (Palmieri et al., 2017).

Different jNCL models were created and used to clarify the pathological mechanisms of CLN3 dysfunction and potential treatments, including cell lines, yeast, amoeba and genetically modified mice (Cotman et al., 2002; Fossale et al., 2004; Gachet et al., 2005; Huber et al., 2016; Mitchison et al., 1999). The most commonly studied and widely accepted jNCL model is a knock-in mouse carrying a deletion of exons 7 and 8 in the *Cln3* gene, representing the same genetic defect found in over 80% of patients (Cotman et al., 2002). The homozygous *Cln3^Δex7/8^* mouse recapitulates many pathological features observed in jNCL patients, with the exception of the early retinal degeneration and vision loss commonly found in jNCL children (Staropoli et al., 2012).

To better simulate the human jNCL condition, we adapted a model of rapid light-induced retinal degeneration in the *Cln3^Δex7/8^* mouse model after introducing the light-sensitive variant of the retinal pigment epithelium-specific 65 kDa protein (RPE65), a genetic modification that is necessary to increase sensitivity to light stress. After finding a jNCL-specific light sensitivity threshold, we tested the effects of the immunomodulatory compound minocycline on microglial reactivity and retinal degeneration. Our data showed that minocycline inhibited microglial responses and significantly delayed retinal degeneration in *Cln3^Δex7/8^* animals, implicating a potential therapeutic use of targeting the immune system in jNCL patients to delay vision loss.

## RESULTS

### CLN3-deficient retinas show only weak retinal thinning but display autofluorescent material in microglia

**Fig. 1.**
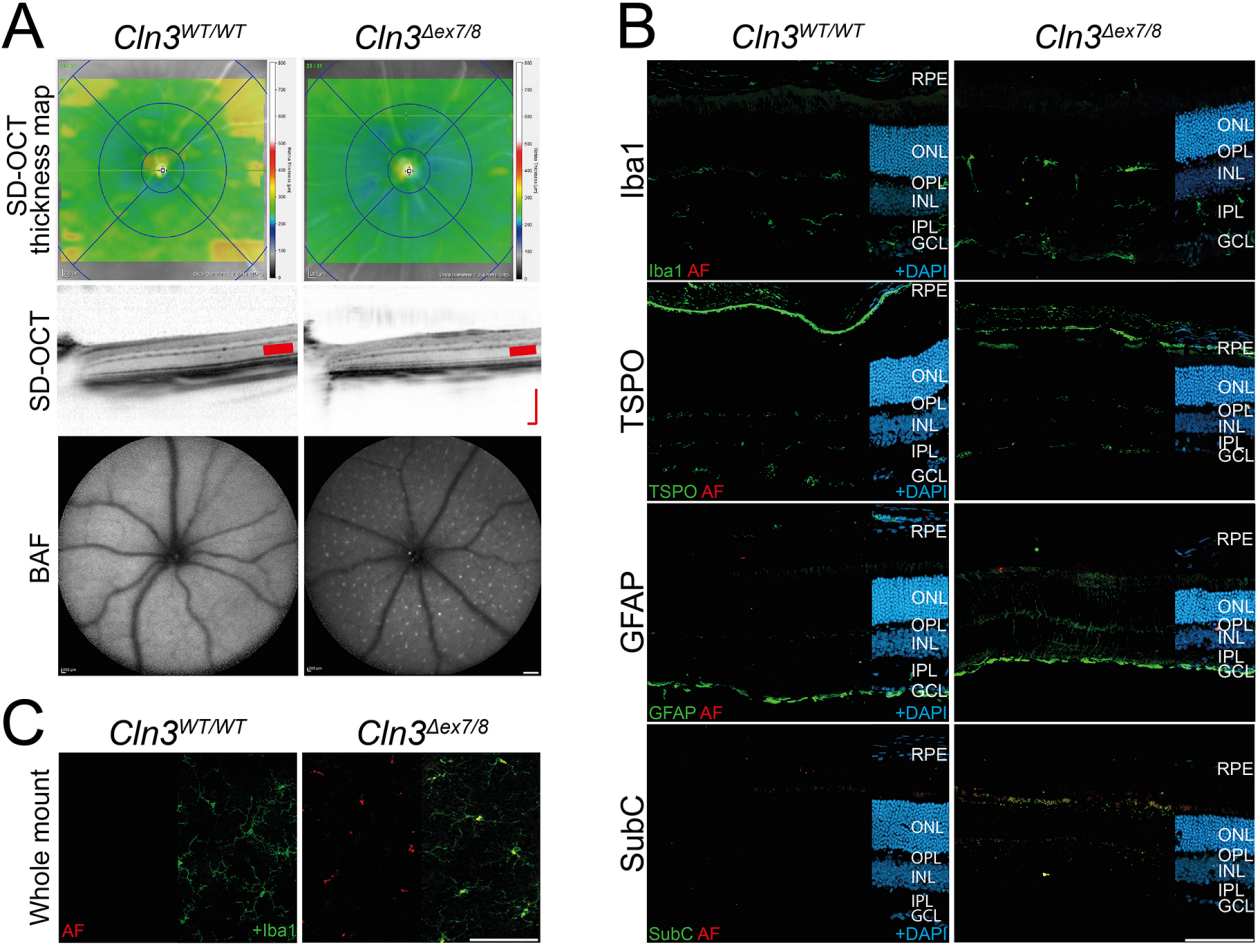
**Retinal phenotype and gliosis in young CLN3-deficient and wild-type mice.** (A) *In vivo* imaging shows only slight thinning of retinas from 8- to 10-week-old CLN3-deficient mice, indicated by the blueish color in retinal SD-OCT thickness maps. No alteration of retinal structure was observed in CLN3-deficient mice compared to wild-type mouse retinas. BAF imaging shows the occurrence of a constant pattern of autofluorescent spots in the fundus of CLN3-deficient mice and the slightly thinner ONL (red bars in SD-OCT scans). Scale bar: 200 µm. (B) Immunohistochemical stainings of retinal cryosections for the microglia marker Iba1 and the gliosis markers TSPO and GFAP revealed no clear difference between CLN3-deficient and wild-type animals. A consistent staining for SubC was present in CLN3-deficient retinas. Scale bar: 100 µm. (C) Iba1-stained retinal flat mounts of CLN3-deficient mice and wild-type mice show normal morphology of microglia and accumulation of autofluorescent storage material only in microglial somata of CLN3-deficient retinas. Scale bar: 100 µm. BAF, BluePeak blue laser autofluorescence; RPE, retinal pigment epithelium; ONL, outer nuclear layer; OPL, outer plexiform layer; INL, inner nuclear layer; IPL, inner plexiform layer; GCL, ganglion cell layer; AF, autofluorescence; TSPO, translocator protein (18 kDa); GFAP, glial fibrillary acidic protein; SubC, mitochondrial ATPase subunit c.

We first examined whether young *Cln3^Δex7/8^* mice carrying the light-sensitive *Rpe65* leucine variant show structural retinal alterations compared to control animals. *In vivo* imaging using spectral domain optical coherence tomography (SD-OCT) identified only a minor thinning of CLN3-deficient retinas compared to wild-type littermates (Fig. 1A, top and middle panels). As accumulation of autofluorescent material is a hallmark of jNCL, we next analyzed the retinal fundus using the BAF (BluePeak Blue laser autofluorescence) method. Evenly distributed autofluorescent spots that were absent in wild-type eyes appeared in the *Cln3^Δex7/8^* retinas (Fig. 1A, bottom panels). To evaluate this in more detail, we analyzed retinal cryosections (Fig. 1B) and flat mounts (Fig. 1C) with immunostainings. We first stained retinal sections with antibodies against Iba1 and translocator protein 18 kDa (TSPO), two markers for ramified and activated microglia, respectively (Lückoff et al., 2016) (Fig. 1B). These data showed the normal appearance of microglia in the plexiform layers and no prominent signs of microglia reactivity in *Cln3^Δex7/8^* retinas compared to littermates (Fig. 1B). Staining for GFAP also revealed only minor features of gliosis (Fig. 1B). We then analyzed mitochondrial ATPase (SubC) as one of the characteristic storage materials found in tissue of jNCL patients. These analyses observed enrichment of SubC within the inner segments of the photoreceptor cells (IS), the inner nuclear layer (INL) and to a lesser extent in the ganglion cell layer (GCL) of CLN3-deficient mice, but not in wild-type littermates (Fig. 1B, bottom panels).

Additional staining of cryosections for cathepsin D (CatD), a marker known to be differentially expressed in CLN3 dysfunction, revealed low CatD expression in the IS, INL and GCL in the aged wild-type retina but increased expression in the aged *Cln3^Δex7/8^* retina with a clustered appearance (Fig. S1A) (Fossale et al., 2004; Golabek et al., 2000). In young animals, higher CatD expression was only noticed in the light-damaged *Cln3^Δex7/8^* retina (Fig. S1B).

Further analyses of autofluorescence and Iba1 localization in retinal flat mounts then showed accumulation of autofluorescent material in microglial somata of CLN3-deficient animals (Fig. 1C). We therefore postulate that microglia somata enriched with lipids appear as autofluorescent spots in the fundus analysis (Fig. 1A).

### CLN3-deficient retinas are highly susceptible to light damage

**Fig. 2.**
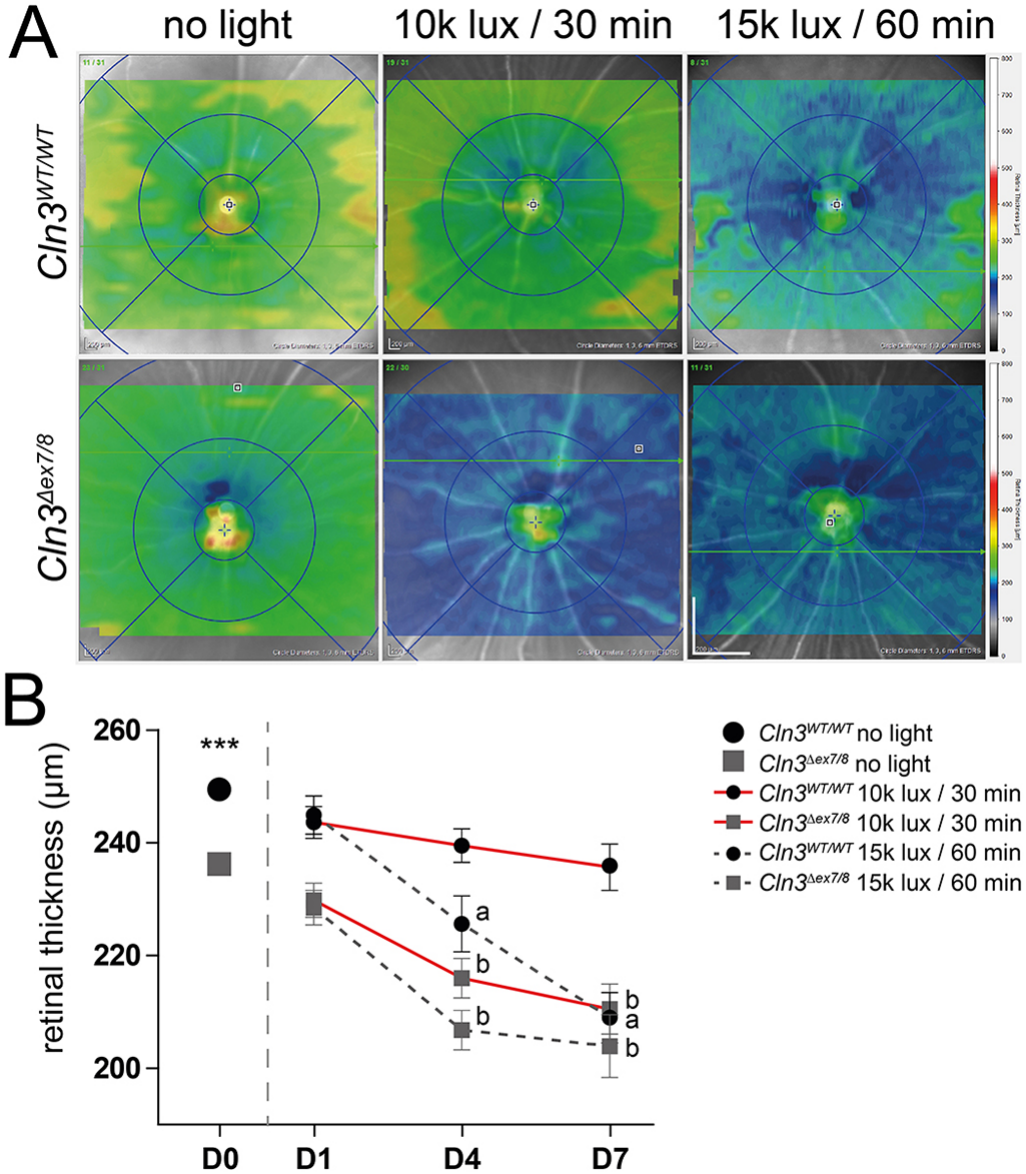
**SD-OCT thickness map shows retinal thinning in ‘low light’ conditions for CLN3-deficient mice and in ‘high light’ conditions for CLN3-deficient and wild-type mice.** (A) Representative SD-OCT thickness maps taken 7 days after light exposure shown for ‘low light’ and ‘high light’ conditions in modified *Cln3^Δex7/8^* and wild-type mice. Scale bar: 1 mm. (B) Analysis of data from unexposed mice showed significant thinning of CLN3-deficient retinas compared to wild-type mice (*n*=43 animals). Quantification of thickness maps from light-exposed animals showed significantly different thinning in the central retinas between CLN3-deficient and wild-type retinas after exposure to ‘low light’ conditions and no significant difference in retinal thickness between both genotypes after ‘high light’ exposure. Data show mean±s.e.m. from four independent experiments (*n*≤10 animals/group) with ***P<0.001. D0, unexposed; D1, 1 day after light exposure; D4, 4 days after light exposure; D7, 7 days after light exposure. ‘a' indicates statistical significance (*P*<0.001) of light-exposed vs unexposed wild-type controls; ‘b' indicates statistical significance (P<0.001) of light-exposed vs unexposed *Cln3^Δex7/8^* mice.

Given the very mild retinal degeneration in young *Cln3^Δex7/8^* mice, we sought to establish a light-damage paradigm that could distinguish CLN3-deficient animals from controls. *Cln3^Δex7/8^* mice and their wild-type littermates, both carrying a light-sensitive *Rpe65* gene variant, were then exposed to two different light intensities, a ‘low light’ condition with 10,000 lux for 30 min and a ‘high light’ condition with 15,000 lux for 60 min. Animals that were not exposed to bright white light served as controls. Retinal thickness was then determined at 1, 4 and 7 days after light exposure using *in vivo* ocular imaging*.* SD-OCT thickness maps of CLN3-deficient mice showed a much stronger retinal thinning under ‘low light’ conditions compared to wild-type mice (Fig. 2A, blue, middle panels). In contrast, ‘high light’ conditions indicated retinal damage in both strains (Fig. 2A, right panels). Quantification of these SD-OCT thickness maps revealed a highly significant retinal thinning (*P*<0.001) for *Cln3^Δex7/8^* animals at a light exposure of 10,000 lux for 30 min at days 4 and 7 (Fig. 2B). In contrast, wild-type mice showed no retinal thinning after exposure to these ‘low light’ conditions (Fig. 2B). As both wild-type and *Cln3^Δex7/8^* animals displayed significant retinal thinning at ‘high light’ conditions, we defined an exposure to 10,000 lux for 30 min as the jNCL-specific threshold for light damage.

**Fig. 3.**
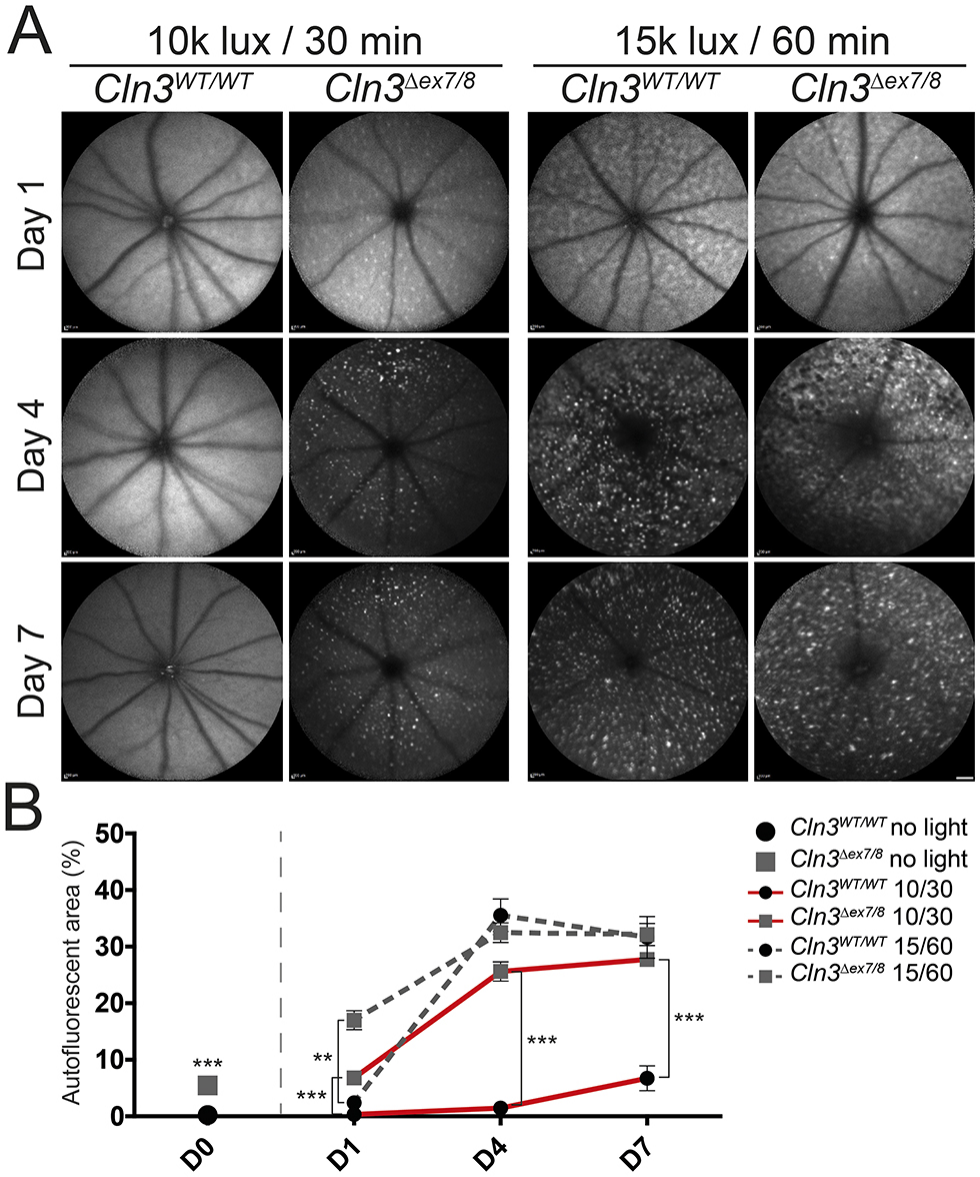
**Accumulation of autofluorescent material in the fundus of white-light-exposed CLN3-deficient and wild-type mice.** (A) BAF imaging shows enriched autofluorescent material in CLN3-deficient retinas after exposure to ‘low light’ conditions, whereas very little autofluorescent material was observed in wild-type animals 7 days after light exposure. Under ‘high light’ conditions, both genotypes show a highly enriched amount of autofluorescent material in the fundus. Scale bar: 1 mm. (B) Comparison of unexposed wild-type and CLN3-deficient retinas shows slight but significant occurrence of autofluorescent material in CLN3-deficient retinas (wild type *n*=32 eyes, *Cln3^Δex7/8^ n*=28 eyes). Quantification of light-exposed data revealed significant difference in autofluorescent storage material of wild-type mice and CLN3-deficient mice under ‘low light’ conditions (*n*≥15 eyes/time point/group). No differences could be observed between both genotypes after ‘high light’ conditions. No significant difference could be shown between 4 days and 7 days after light exposure (*n*≥11 eyes/time point/group). Data show mean±s.e.m. from four independent experiments with **P<0.01 and ***P<0.001. The lux value (×1000) and length of exposure (30 or 60 min) is shown in the key. D0, unexposed; D1, 1 day after light exposure; D4, 4 days after light exposure; D7, 7 days after light exposure.

We then evaluated whether exposure to bright white light leads to changes in the amount of autofluorescent material (Fig. 3). Storage material was observed in CLN3-deficient retinas under both ‘low light’ and ‘high light’ conditions (Fig. 3A, left panels), whereas autofluorescent material appeared in wild-type retinas only under ‘high light’ conditions (Fig. 3A, right panels). Quantification of the autofluorescent area revealed significant differences between *Cln3^Δex7/8^* mice and control animals when applying ‘low light’ conditions at day 4 and day 7 (Fig. 3B). No significant differences were identified between wild-type and CLN3-deficient retinas under ‘high light’ conditions.

### Microgliosis and accumulation of autofluorescent material in CLN3-deficient retinas at ‘low light’ conditions

**Fig. 4.**
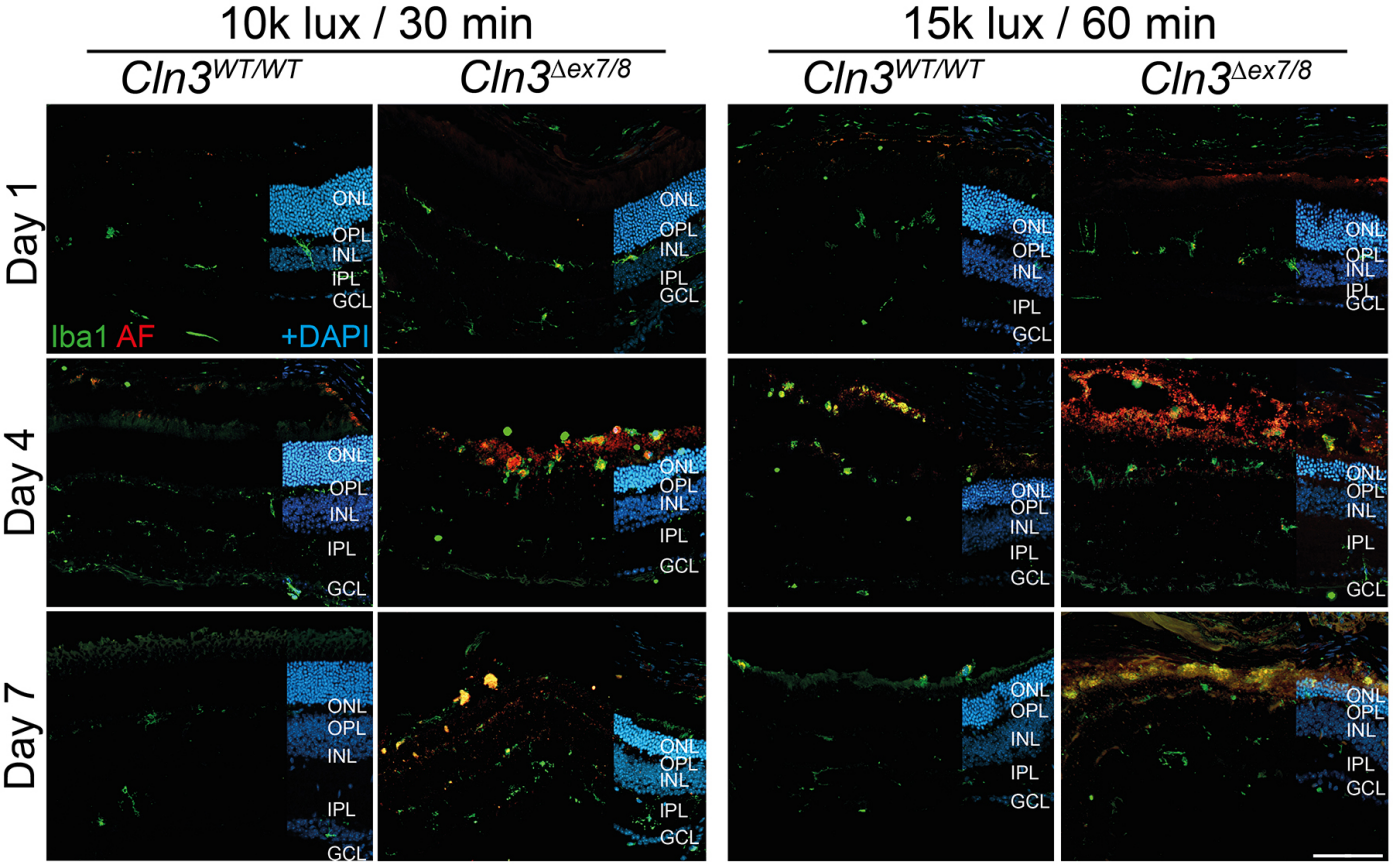
**Microgliosis in white-light-exposed CLN3-deficient mice.** Iba1-positive cells were located in the plexiform layers of wild-type mice exposed to ‘low light’ conditions. Microglial protrusions recognizing the beginning of cell death in the ONL are visible at day 1. Microglial migration towards the subretinal space was visible at 4 and 7 days after light exposure in CLN3-deficient retinas after ‘low light’ and ‘high light’ conditions, and for wild-type retinas only after ‘high light’ conditions. Scale bar: 100 µm. AF, autofluorescence; ONL, outer nuclear layer; OPL, outer plexiform layer; INL, inner nuclear layer; IPL, inner plexiform layer; GCL, ganglion cell layer.

**Fig. 5.**
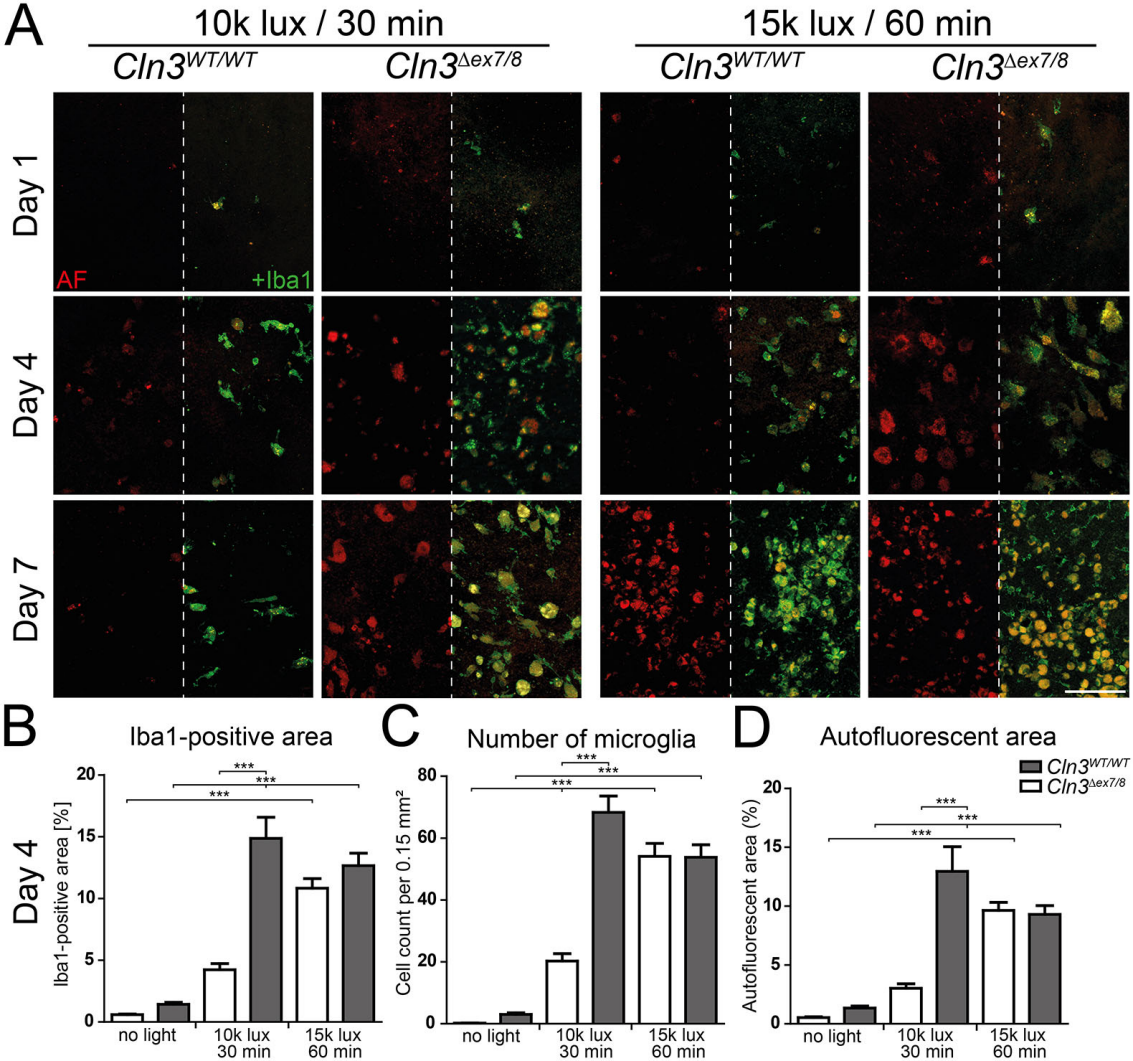
**Microglial accumulation of autofluorescent material in white light-exposed CLN3-deficient mice.** (A) Some Iba1-positive cells are already observable in the subretinal space 1 day after light exposure in CLN3-deficient retinas exposed to ‘low light’ and ‘high light’ conditions as well as in wild-type mice after exposure to ‘high light’ conditions. Only a few microglia are found in the subretinal space of wild-type mice after ‘low light’ conditions at all three time points. An increased amount of autofluorescent material was present 4 and 7 days after light exposure in CLN3-deficient retinas under ‘low light’ conditions and for both genotypes under ‘high light’ conditions. Scale bar: 100 µm. (B-D) Quantification of the Iba1-positive area (B), the number of microglial (C) and the area covered by autofluorescent material (D) show highly significant differences between CLN3-deficient and wild-type mice treated with ‘low light’ conditions. Data show mean±s.e.m. (*n*=3 mice/group) from two independent experiments with ***P<0.001. AF, autofluorescence.

We next analyzed microgliosis in retinas exposed to either light condition. Iba1 staining of cryosections showed microglia migration towards the outer retina starting at day 4 in ‘low light’-exposed CLN3-deficient mice but not in wild-type mice (Fig. 4, left panels). Accumulation of autofluorescent material was also detected at these time points, which are known as peaks of microgliosis (Lückoff et al., 2016). Microgliosis was seen in both wild-type and CLN3-deficient retinas under ‘high light’ conditions (Fig. 4, right panels). Concurrent Iba1-stained flat mounts verified a higher number of microglia in the subretinal space of CLN3-deficient animals exposed to ‘low light’ conditions, compared to controls (Fig. 5A, left panels). Quantification then showed an increase in the Iba1-positive area (Fig. 5B), the total number of microglial cells (Fig. 5C) and the autofluorescent area (Fig. 5D) in battenin-deficient retinas compared to wild-type tissue. No significant differences were observed between wild-type and CLN3-deficient retinas at ‘high light’ conditions (Fig. 5A, right panels; Fig. 5B-D). This difference in gliosis between CLN3-deficient and wild-type mice exposed to ‘low light’ conditions was also seen with a stronger expression of TSPO (Fig. S2) and GFAP (Fig. S3) in cryosections of *Cln3^Δex7/8^* animals.

To assess the expression of pro-inflammatory mediators, we then measured gene expression of the microgliosis markers activated microglia/macrophage whey acidic protein (AMWAP), TSPO and CD68 using quantitative real-time PCR (qRT-PCR) 4 days after light exposure (Fig. S4). All three markers showed a significant increase in CLN3-deficient retinas exposed to ‘low light’ conditions compared to wild-type retinas. Notably, upregulation of TSPO and CD68 was even detected in CLN3-deficient retinas not exposed to light (Fig. S4B,C).

Further analysis of SubC revealed increased amounts in CLN3-deficient retinas, whereas wild-type retinas only showed SubC deposits at ‘high light’ conditions at day 7 (Fig. S5). Staining of CatD in retinal cryosections of ‘low light’-exposed *Cln3^Δex7/8^* mice and ‘high light’-exposed wild-type mice revealed a cluster-like appearance similar to the CatD distribution in the aged CLN3-deficient animals (Fig. S1B, bottom images).

### Transcriptomic effects of light stress in CLN3-deficient retinas mirror disease-specific patterns

We next evaluated whether the ‘low light’ exposure conditions elicit jNCL-specific changes in *Cln3^Δex7/8^* mice that are different from wild-type responses. Therefore, we performed a global gene expression analysis using DNA microarrays. Gene expression dynamics inspector (GEDI) analysis that creates self-organizing maps by ‘gestalt’ recognition (Eichler et al., 2003) showed prominent but different clusters of light-dependent changes in wild-type mice (Fig. S6A) and *Cln3^Δex7/8^* mice (Fig. S6B). In *Cln3^Δex7/8^* retinas, ‘low light’ conditions influenced two major gene clusters that were either reduced (Fig. S6B, top rectangles) or induced (Fig. S6B, bottom rectangles). An in-depth analysis of the transcripts in these clusters revealed 522 significantly upregulated and 623 downregulated genes in light-triggered CLN3-deficient retinas (Fig. S7A). Further comparisons of these dysregulated genes in light-damaged *Cln3^Δex7/8^* mice with the light-regulated transcriptome of wild-type animals revealed 224 upregulated and 55 downregulated genes specifically present in CLN3-deficient retinas (Fig. S7B). Pathway analysis showed that these CLN3-specific transcriptomic changes were associated with different biochemical and cellular processes. However, the majority of the upregulated genes (39%) were associated with immune responses and more specifically were pro-inflammatory (Fig. S7C). The genes downregulated in *Cln3^Δex7/8^* retinas under ‘low light’ conditions were found in several different pathways (Fig. S7B) and only a minor fraction was immune-related (Fig. S7C).

### Minocycline therapy prevents retinal thinning and accumulation of autofluorescent material

**Fig. 6.**
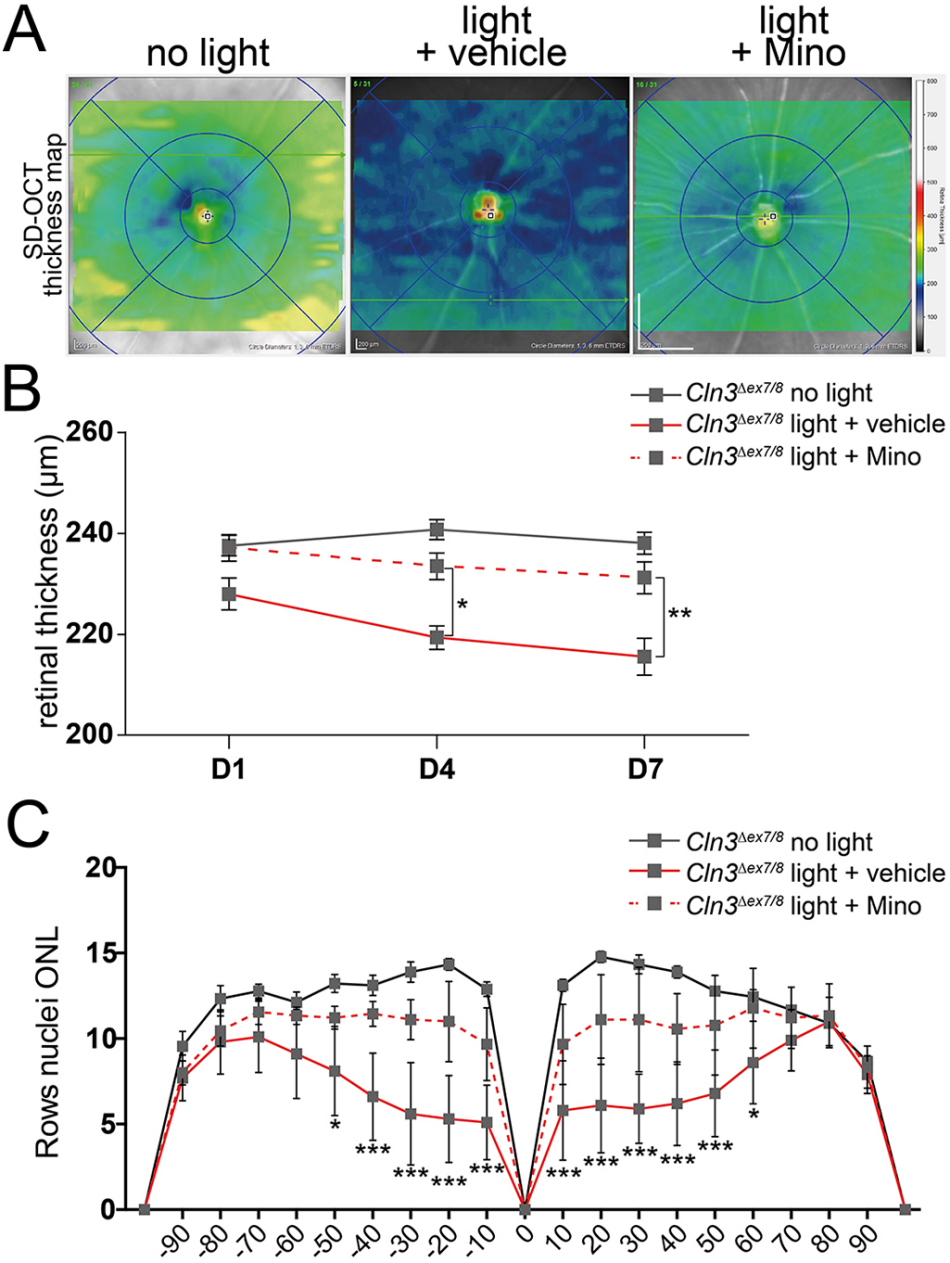
**Minocycline prevents retinal thinning of CLN3-deficient mice after white light exposure.** (A) Representative SD-OCT thickness maps reveal less retinal thinning (green) in minocycline-treated mice compared to vehicle-treated CLN3-deficient mice (blue). (B) Quantification of central retinal thickness shows significant reduction in retinal thinning in minocycline-treated mice after exposure to white light (*n*≥9 animals/ group). (C) Counting photoreceptor cell nuclei in DAPI-stained cryosections shows the same tendency as the thickness maps (*n*≥9 eyes/group). The *x*-axis shows the distance from the optic nerve head in %. Data show mean±s.e.m. from three independent experiments with *P<0.05, **P<0.01, ***P<0.001 light exposed+vehicle versus light exposed+minocycline. D1, 1 day after light exposure; D4, 4 days after light exposure; D7, 7 days after light exposure; Mino, minocycline.

We have previously shown that immunomodulation with minocycline has beneficial effects on retinal degeneration in the light-damage paradigm (Scholz et al., 2015). To test whether the susceptibility of CLN3-deficient mice to ‘low light’ conditions can be reduced by this form of therapy, animals were treated by intraperitoneal injection of minocycline (45 mg/kg body weight) or 0.9% sodium chloride solution daily, starting 1 day before light exposure. The SD-OCT thickness maps showed a greenish color for minocycline-treated animals compared to the blueish color from vehicle-treated animals (Fig. 6A). Quantification revealed a significant preservation of retinal thickness for minocycline-treated animals compared to vehicle-treated mice (Fig. 6B). These findings were confirmed by counting the rows of photoreceptor cell nuclei in DAPI-stained cryosection panoramas. The resulting spidergram analysis showed a significant preservation of photoreceptor cell nuclei in the central parts of the retina after minocycline treatment compared to vehicle-treated CLN3-deficient mice (Fig. 6C).

**Fig. 7.**
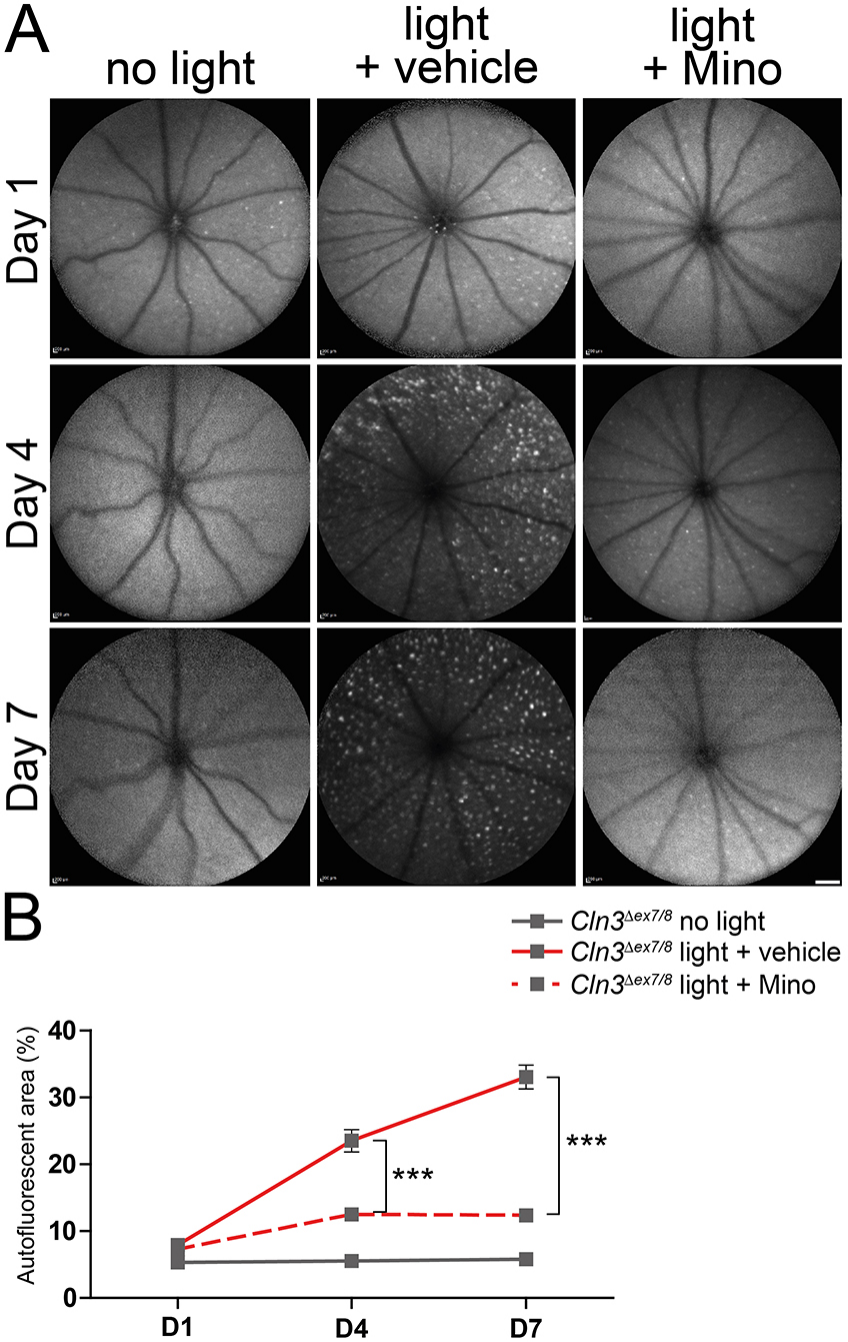
**Less autofluorescent material in the fundus of CLN3-deficient mice after minocycline administration.** (A) BAF imaging shows a clear reduction of autofluorescent material at 1, 4 and 7 days after light exposure in minocycline-treated CLN3-deficient animals compared to vehicle-treated mice. Scale bar: 1 mm. (B) Quantification of the area covered by autofluorescent material shows a significant decrease at 4 and 7 days after light exposure in minocycline-treated animals (*n*≥7 eyes/group). Data show mean±s.e.m. from three independent experiments with ***P<0.001. D1, 1 day after light exposure; D4, 4 days after light exposure; D7, 7 days after light exposure; Mino, minocycline.

Furthermore, minocycline treatment prevented the accumulation of autofluorescent material in the retina (Fig. 7A). A significantly lower area of autofluorescent material was detected at 4 days and 7 days after light exposure in minocycline-treated retinas compared to vehicle-treated retinas (Fig. 7B). This suggests that minocycline efficiently prevents the light-induced photoreceptor loss in *Cln3^Δex7/8^* animals.

### Minocycline therapy prevents microglial reactivity in CLN3-deficient retinas

**Fig. 8.**
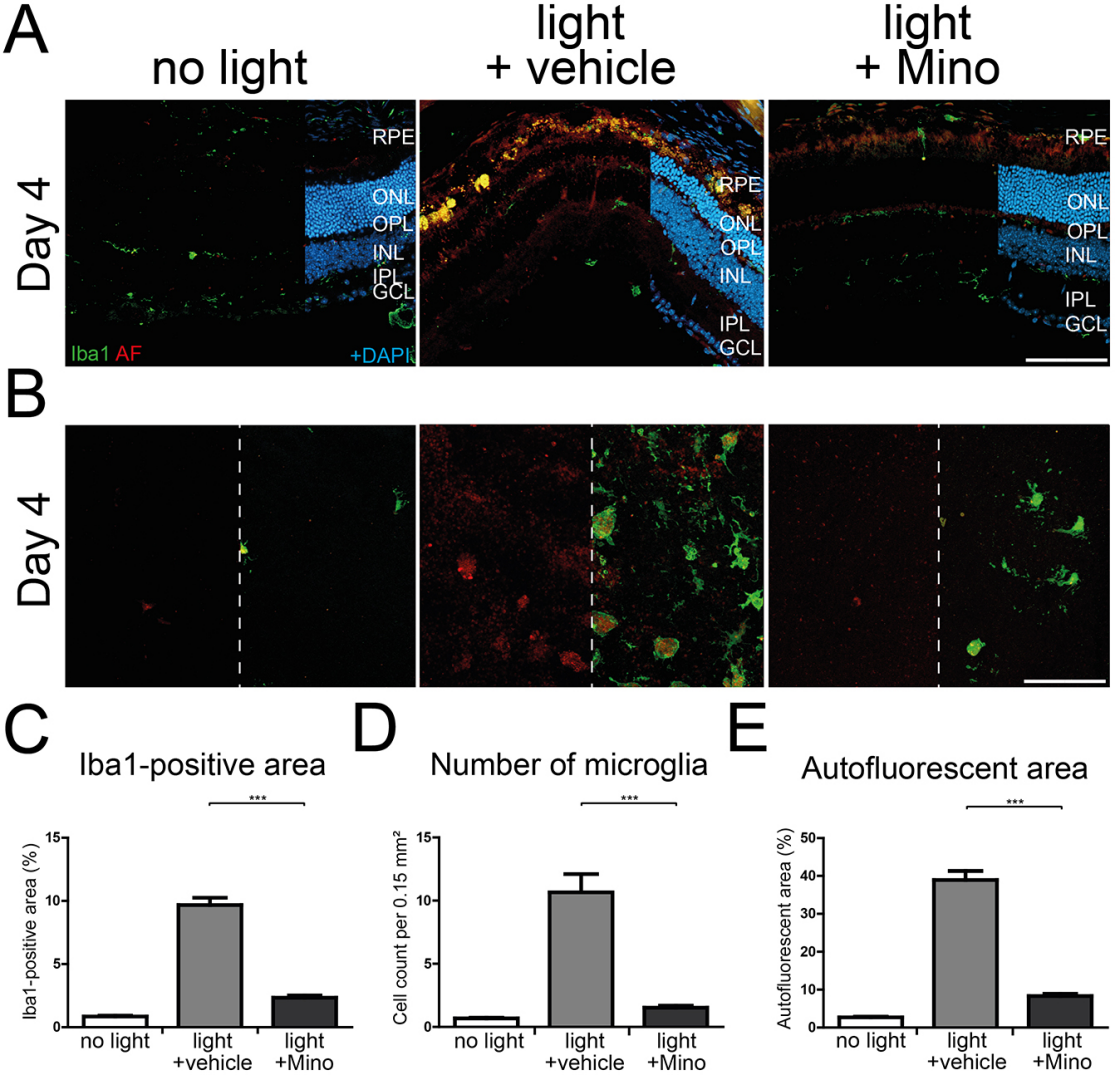
**Lower microglial activity in minocycline-treated CLN3-deficient retinas after white-light exposure.** (A) Iba1-stained retinal cryosections show less microglial migration towards the subretinal space 4 days after light exposure when mice are treated with minocycline during the white-light exposure regimen compared to vehicle-treated animals. Scale bar: 100 µm. (B) Less autofluorescent material in microglial cells is present 4 days after light exposure in minocycline-treated CLN3-deficient animals compared with vehicle-treated animals. A high amount of autofluorescent material and many microglial cells were present in the subretinal space of vehicle-treated animals at day 4. Scale bar: 100 µm. (C-E) Quantification of the Iba1-positive area (C), number of microglial cells (D) and area covered by autofluorescent material (E) showed significant reductions after minocycline administration in comparison to vehicle-treated animals 4 days and 7 days after light exposure. Data show mean±s.e.m. (*n*=24 pictures/group) from two independent experiments with ***P<0.001. RPE, retinal pigment epithelium; ONL, outer nuclear layer; OPL, outer plexiform layer; INL, inner nuclear layer; IPL, inner plexiform layer; GCL, ganglion cell layer; Mino, minocycline.

We next studied the effects of minocycline on microgliosis in light-exposed CLN3-deficient retinas. A strong microglial migration towards the subretinal space was observed in vehicle-treated retinas (Fig. 8A, middle panels). In contrast, only a minor migration of microglia was seen in minocycline-treated retinas (Fig. 8A, right panels). Additionally, Iba1-stained flat mounts showed reduced microglial occurrence in the subretinal space as well as a lower amount of autofluorescent material in the minocycline treatment group (Fig. 8B). Quantification of Iba1-positive area, number of microglial cells and the area of autofluorescent material showed significant reduction of all three analysis parameters in mice treated with minocycline compared to vehicle treatment (Fig. 8C-E).

In accordance with the data from microglia migration, lower expression of TSPO and GFAP was present in cryosections of minocycline-treated *Cln3^Δex7/8^* mice compared to the vehicle-treated group (Fig. S8A,B). Furthermore, the increased accumulation of SubC observed in CLN3-deficient retinas after light exposure was reduced when animals were treated with minocycline (Fig. S8C).

Retinal qRT-PCR analysis of the pro-inflammatory markers AMWAP, CCL2 (CC-chemokine ligand 2), CD68, IL1β (interleukin 1β), TNFα (tumor necrosis factor α) and TSPO (translocator protein 18 kDa) measured 4 days after light exposure showed a significantly lower expression in minocycline-treated animals compared to vehicle-treated animals (Fig. S9).

### Minocycline downregulates pro-inflammatory gene clusters

To evaluate whether minocycline treatment has an influence on the upregulated genes that were specifically found in light-damaged CLN3-deficient retinas, microarray datasets from light-exposed and minocycline-treated *Cln3^Δex7/8^* mice were compared to light-exposed and vehicle-treated *Cln3^Δex7/8^* mice. The GEDI maps showed lower expression of gene clusters arising under light-triggered conditions in CLN3-deficient mice (Fig. S6B, bottom right rectangles). Among these genes, 22 genes were significantly downregulated by minocycline administration (Fig. S10A) and the majority was associated with immune responses pathways (Fig. S10B). Finally, qRT-PCR was used to validate three immune-related genes that were specifically light-induced in *Cln3^Δex7/8^* retinas and counter-balanced by minocycline. Apolipoprotein D (*ApoD*), interferon regulatory factor 7 (*Irf7*) and Z-DNA binding protein 1 (*Zpb1*) showed the expected patterns of regulation, similar to the profiles identified by DNA-microarrays (Fig. S11).

## DISCUSSION

Chronic microglia reactivity and expression of neurotoxic substances are detrimental for the brain and the retina (Hanisch and Kettenmann, 2007; Langmann, 2007; Zhao et al., 2015). Since immune responses in the CNS occur in jNCL models at higher ages (Pontikis et al., 2004), we here analyzed microglial behavior in young CLN3-deficient mice. We saw no clear characteristics of inflammation and only a slight thinning of the retina in 8- to 10-week-old mice. Significant retinal thinning was present in another jNCL mouse, the *Cln3^−/−^* model, at 18 months (Groh et al., 2014). These CLN3-deficient mice also showed autofluorescent material in the fundus, verifying our data. Furthermore, accumulation of autofluorescent material was detected in neuronal tissue of jNCL patients (Bensaoula et al., 2000; Cotman et al., 2002). Here, we showed that microglia in *Cln3^Δex7/8^* animals were correctly located in the plexiform layers, showed a ramified morphology, and had low expression of TSPO and GFAP. These findings are in line with the low neuronal immune responses described in another publications (Pontikis et al., 2004). Our data are in contrast to *in vivo* and *ex vivo* experiments describing altered brain microglia morphology and impaired behavior (Parviainen et al., 2017; Xiong and Kielian, 2013). A further hallmark of jNCL pathology is the accumulation of autofluorescent storage material composed of SubC, lipoproteins and glycoproteins, assumed to play the major role in neuronal cell death (Palmer et al., 1992). Here, accumulation of SubC was shown in cryosections of CLN3-deficient mice in all retinal layers. The distribution pattern of SubC stainings in our *Cln3^Δex7/8^* animals was very similar to the pattern of autofluorescent material in *Cln3^−/−^* mice (Seigel et al., 2002).

Most other mouse models of NCL, including the *Cln5*^−/−^ and *Cln6*^nclf^ mouse, which mimics late-infantile NCL, display prominent retinal degeneration and microglia reactivity at younger ages (Bartsch et al., 2013; Leinonen et al., 2017; Mirza, 2013). Therefore, a major goal of this study was to experimentally modify the *Cln3^Δex7/8^* model for jNCL so that it showed an earlier retinal phenotype, comparable to the human situation. Exposure to bright white light is a widely used model for retinal degeneration and microglial activation (Grimm and Remé, 2013). Therefore, it was necessary to genetically introduce a specific Rpe65 variant leading to light sensitivity of *Cln3^Δex7/8^* mice by crossing with BALB/c animals (Samardzija et al., 2006). The resulting modified CLN3-deficient mice showed a higher susceptibility to white-light exposure than the original strain and, importantly, also compared to wild-type animals that were not susceptible to light exposure at 10,000 lux for 30 min. Under these ‘low light’ conditions, the genetically modified CLN3-deficient mice displayed a strong thinning of retinal layers and showed typical hallmarks for microgliosis. *In vivo* imaging and stainings also detected increased amounts of autofluorescent material in ‘low light’-exposed *Cln3^Δex7/8^* animals, potentially reflecting increased autofluorescence observed in the fundus of early-stage jNCL patients (Kelly et al., 2009).

The question arises of whether the ‘low light’-induced early retinal degeneration in *Cln3^Δex7/8^* mice recapitulated the retinal changes in jNCL patients. Our data support this hypothesis in several ways. First, besides the accumulation of SubC as discussed above, a dysregulation of the lysosomal enzyme CatD has been connected with the absence of CLN3 (Cárcel-Trullols et al., 2017; Fossale et al., 2004; Golabek et al., 2000; Kyttälä et al., 2006). Indeed, here we showed enrichment of CatD in ‘low light’-triggered retinal degeneration in young *Cln3^Δex7/8^* mice that was normally only present in aged *Cln3^Δex7/8^* mice. Second, the transcriptomic analysis showed light-dependent dysregulation of a high number of genes in *Cln3^Δex7/8^* retinas that were not regulated in wild-type animals. We found a strong effect of retinal CLN3 deficiency on immune response pathways that was also previously noticed in total eye and cerebellum transcripts from young *Cln3*^−/−^ mice (Chattopadhyay et al., 2004; Brooks et al., 2003). Of particular interest were those genes that were upregulated in light-triggered *Cln3^Δex7/8^* animals compared to light-damaged wild-type mice and in turn downregulated with minocycline treatment. *Irf7* and *Zbp1*, two genes that regulate immune responses and are associated with neuronal cell loss, are highly upregulated in CLN3-deficient and light-triggered mice, and are examples for a possible overshooting immune reaction already described for CLN3-deficient primary microglia (DeFilippis et al., 2010; Gao et al., 2017; Mustafi et al., 2012; Xiong and Kielian, 2013).

Minocycline is an approved clinical antibiotic drug that is also known to dampen microglial reactivity and prevent photoreceptor loss in several mouse models of neuronal degeneration (Du et al., 2001; Peng et al., 2014; Scholz et al., 2015; Zhao et al., 2011). Consistent with these reports, minocycline showed a protective effect on light-triggered microglial activation and retinal thinning in our modified CLN3-deficient mouse model. *In vivo* imaging in combination with immunohistochemical stainings showed a preservation of retinal thickness when CLN3-deficient mice were treated with minocycline during white-light damage. Additionally, minocycline administration reduced the accumulation of autofluorescent material in the subretinal space. This is probably due to reduced neuronal cell death and therefore less cellular debris. We found less microgliosis and lower expression of CD68 and IL-1β, which is in line with the published effects of minocycline in an ischemia-reperfusion model of retinal degeneration (Ahmed et al., 2017). In our CLN3-deficient mice, minocycline suppressed expression of the pro-inflammatory mediators AMWAP, TSPO and CCL2, which was previously demonstrated for wild-type mice under high-light conditions (Scholz et al., 2015).

The decreased expression of pro-inflammatory mediators in combination with preservation of photoreceptor cells and reduction of storage material leads to the assumption that minocycline administration mediates its effects mainly via immunomodulation. However, a study with orally treated CLN6-deficient sheep, a model for the late-infantile variant of NCL, with minocycline, could not dampen neuroinflammation in the brain (Kay and Palmer, 2013). The species differences and different forms of NCL may potentially explain the treatment success in our modified mouse retinal stress model and the disappointing findings from CLN6 South Hampshire sheep.

## MATERIALS AND METHODS

### Mouse handling

*Cln3^Δex7/8^* mice on a C57BL/6J background were a generous gift from S. Cotman (Cotman et al., 2002). To introduce the light-sensitive Rpe65 leucine variant in the strain, the mice were first crossed with wild-type BALB/c and then with C57BL/6J mice for five generations to obtain a homogenous genetic background. Resulting *Cln3^Δex7/8^* mice, carrying the leucine variant of RPE65 on a C57BL/6J background, were genotyped to determine the presence of the *Cln3^Δex7/8^* knock-in allele as previously described (Fossale et al., 2004). For the *Rpe65* variant, a PCR fragment of 674 bp length was generated using the following primers: Rpe65_pos450_F (5′-CACTGTGGTCTCTGCTATCTTC-3′) and Rpe65_pos450_R (5′GGTGCAGTTCCATTCAGTT-3′) with PCR cycling conditions of 95°C for 30 s, 56°C for 30 s and 72°C for 35 s, repeated for 34 cycles. Amplicons were digested using *Mwo*I to detect a specific restriction site at position 450, resulting in two fragments, of 236 and 437 bp, for the leucine variant or a 674 bp undigested fragment for the methionine variant (Wenzel et al., 2001). Mice were maintained in an air-conditioned environment with 12-h light-dark schedule with a maximum light intensity of 150 lux inside the cages. Water and food were given *ad libitum*. For this study, 8- to 10-week-old male and female animals were used. All experimental procedures were in compliance with the German law on animal protection and the ARVO Statement for the Use of Animals in Ophthalmic and Vision Research. The animal protocols used in this study were reviewed and approved by the governmental body responsible for animal welfare in the state of Nordrhein-Westfalen (Landesamt für Natur, Umwelt und Verbraucherschutz Nordrhein-Westfalen, Germany; approval Nr 84-02.04.2017.A113).

### Light exposure and minocycline administration

Wild-type littermates and *Cln3^Δex7/8^* mice were dark-adapted for 16 h before light exposure. To test low-light and high-light conditions, pupils were dilated with 2.5% phenylephrin and 1% tropicamide under dim red light and the mice were placed into aluminum-foil-coated cages. A light intensity of 10,000 lux for 30 min for low-light conditions and a light intensity of 15,000 lux for 60 min for high-light conditions were used, respectively. Mice of both genotypes without light exposure served as controls. Then, mice were then kept in dark-reared conditions overnight. For the therapy study with minocycline only, *Cln3^Δex7/8^* mice and low-light conditions were used. Minocycline (45 mg/kg body weight dissolved in 0.9% NaCl solution, Sigma) was injected intraperitoneally starting 1 day before light exposure and then daily until 1 day before the final experiment. *Cln3^Δex7/8^* mice were injected with the same volume and frequency of 0.9% NaCl solution as a solvent control.

### *In vivo* imaging using SD-OCT and BAF

SD-OCT and BAF images were taken at 1, 4 and 7 days after light exposure. After anesthetizing the mice by intraperitoneal injection of Rompun (10 mg/kg body weight) and Ketavet (100 mg/kg body weight), the pupils were dilated with 2.5% phenylephrin and 1% tropicamide. SD-OCT was performed on both eyes with a Spectralis™ HRA+OCT device (Heidelberg Engineering) to evaluate structural changes. Retinal thickness was analyzed using SD-OCT thickness maps calculated by the Heidelberg Eye Explorer Software. A mean for each eye was determined from the four quarters (circle diameter 3 ETDRS) around the optic nerve head, representing the average retinal thickness (µm) in a certain field. BAF imaging was conducted at day 1, 4 and 7 to assess autofluorescent material using a Spectralis™ HRA+OCT device (Heidelberg Engineering). Three images per condition were analyzed for quantification.

### Immunohistochemistry and image analysis

For immunohistochemical analysis, eyes were enucleated at day 1, 4 and 7, and fixed in 4% formaldehyde for 2 h at room temperature. Eyes were dissected for retinal flat mounts or transferred into 30% sucrose overnight at room temperature (RT) before embedding in optimal cutting temperature (OCT) compound for cryosectioning. Sections of 12 µm thickness were rehydrated with PBS, blocked with dry milk solution and incubated with primary antibody solution. Flat mounts were permeabilized with 2.5% Triton X-100 and 2.5% Tween-20, blocked with dry milk solution and incubated in primary antibody solution. Specific antibodies against Iba1 (1:500, Wako Chemicals, Neuss, Germany; 1:500 donkey-anti goat, Abcam, Cambridge, UK), TSPO (1:250, Abcam), GFAP (1:400, Sigma, MO, USA) and SubC (1:250, Abcam) were used overnight at 4°C. Afterwards, flat mounts and sections were incubated with the appropriate secondary antibodies conjugated to Alexa Fluor 488 (Jackson Immuno-Research, West Grove, PA, USA) at RT for 1 h. Cryosections were counterstained with 4′,6-diamidino-2-phenylindol (DAPI). All samples were mounted with DAKO fluorescent mounting medium (Dako Deutschland GmbH, Hamburg, Germany) and analyzed with an AxioImager.M2 plus ApoTome2 microscope (Carl Zeiss). Iba1-positive area, number of microglia and area of autofluorescent material was evaluated by ImageJ. For this purpose, 24 images (20× magnification) of each eye were taken of three animals from three independent experiments and analyzed using FIJI software.

### qRT-PCR

RNA was isolated from freshly dissected retinal tissue and transcribed to cDNA. A total of 25 ng cDNA was amplified using the RevertAid™ H-Minus First Strand cDNA Synthesis Kit (Fermentas, Germany). Amplification of 40 ng cDNA was performed on an ABI7900HT machine (Applied Biosystems, Carlsbad, CA, USA). Reaction mixtures contained 2.5 µl sample, 1× Fast Start Universal Probe Master (Rox; Roche Applied Science, Switzerland), 100 nM of primers and 0.125 μl of dual-labeled UPL probe (Roche). A setting of the following reaction parameters were used: 10 min 95°C hold, 40 cycles of 15 s 95°C melt, followed by 1 min 60°C anneal/extension. Used primers and probes were as followed: *AMWAP*, forward primer 5′-TTTGATCACTGTGGGGATGA-3′, reverse primer 5′-ACACTTTCTGGTGAAGGCTTG-3′, probe #1; *ApoD*, forward primer 5′-AATTTCCATCTTGGGAAATGC-3′, reverse primer 5′-GGATCTTCTCAATTTCGTACCATC-3′, probe #63; *Atp5b*, forward primer 5′-GGCACAATGCAGGAAAGG-3′, reverse primer 5′-TCAGCAGGCACATAGATAGCC-3′, probe #77; *CCL2*, forward primer 5′-CATCCACGTGTTGGCTCA-3′, reverse primer 5′-GATCATCTTGCTGGTGAATGAGT-3′, probe #62; *CD68*, forward primer 5′-CTCTCTAAGGCTACAGGCTGCT-3′, reverse primer 5′-TCACGGTTGCAAGAGAAACA-3′; probe #27, *IL1β*, forward primer 5′-TGTAATGAAAGACGGCACACC-3′, reverse primer 5′-TCTTCTTTGGGTATTGCTTGG-3′; probe #78, *Irf7*, forward primer 5′-CTTCAGCACTCCCTTCCGAGA-3′, reverse primer 5′-TGTAGTGTGGTGACCCTTGC-3′; probe #25, *Zbp1*, forward primer 5′-GCTGTGAGCATGGCAGAA-3, reverse primer 5′-CACCTGCAGGATCTTTTGCT-3′; probe #99, *Tnfα*, forward primer 5′-CTGTAGCCCACGTCGTAGC-3′, reverse primer 5′-TCTTCTTTGGGTATTGCTTGG-3′; *TSPO*, forward primer 5′-ACTGTATTCAGCCATGGGGTA-3′, reverse primer 5′-ACCATAGCGTCCTCTGTGAAA-3′, probe #33. Measurements were performed in technical duplicates. *ATP5B* expression was used as reference gene and resulting values were calculated using the ΔΔCt method for relative quantification.

### DNA microarrays and bioinformatic data analysis

Duplicate microarrays were carried out with two independent RNAs and procedures involving preparation and labeling of cRNA, hybridization to Affymetrix 430 2.0 mouse genome arrays, washing and scanning were performed according to the standard protocol. The cut-off applied for genes considered to be differentially expressed was a log2 fold change of ≥2 or ≤−2 and a *P*<0.05. Integrative analysis of genome-wide expression activities was performed with the Gene Expression Dynamics Inspector (GEDI), a MatLab (MathWorks, Natick, MA, USA) freeware program, which uses self-organizing maps (SOMs) to translate high-dimensional data into a 2D mosaic (Eichler et al., 2003). Each tile of the mosaic represents an individual SOM cluster and is color-coded to represent high or low expression of the cluster's genes, thus identifying the underlying pattern. Functional annotation of transcripts was performed using Bibliosphere pathway edition (Genomatix). DNA-microarray data were deposited in the Gene Expression Omnibus (GEO) (GSE118664).

### Statistical analyses

Statistical analysis was performed using GraphPad Prism 6. For analysis of two groups, an unpaired Student's *t*-test was performed. To compare more than two groups, one-way ANOVA followed by Tukey's post-test was used. All values are presented as mean±s.e.m. *P*-values ≤0.05 were considered as significant.

## Supplementary Material

Supplementary information

First Person interview
