# Supplementary material for: Immunomodulation with minocycline rescues retinal degeneration in juvenile neuronal ceroid lipofuscinosis mice highly susceptible to light damage
Source: Dis Model Mech. 2018 Sep 5;11(9):dmm033597. doi: 10.1242/dmm.033597 (PMC6176999; doi:10.1242/dmm.033597)
Supplement: Supplementary information [file dmm-11-033597-s1.pdf]

FIRST PERSON

First person – Katharina Dannhausen

First Person is a series of interviews with the first authors of a selection of papers published in Disease Models & Mechanisms, helping early-career researchers promote themselves alongside their papers. Katharina Dannhausen is first author on 'Immunomodulation with minocycline rescues retinal degeneration in juvenile neuronal ceroid lipofuscinosis mice highly susceptible to light damage', published in DMM. Katharina is a postdoc in the lab of Prof. Dr Thomas Langmann at the University of Cologne, Cologne, Germany, investigating the interaction between the immune system and CLN3-deficient neuronal cells in the retina.

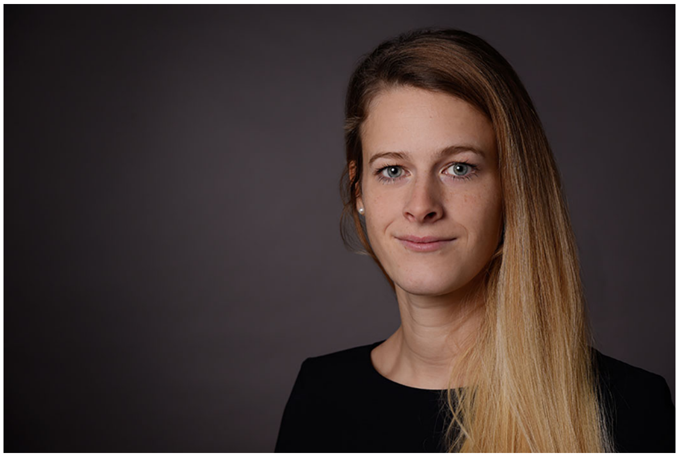

Katharina Dannhausen

How would you explain the main findings of your paper to non-scientific family and friends?

Juvenile neuronal ceroid lipofuscinosis (jNCL) is a rare but very severe disease affecting children at school age. Beginning with blindness, those kids lose previously acquired skills, exhibit seizures and become bedridden until they die in their late 20s. The reason for these symptoms is a malfunction of CLN3, a protein of so far unknown function leading to neuronal cell death in the retina and brain. Neuronal cell death is usually accompanied by a strong activation of the immune system, leading to additional neuronal damage. The most frequently used jNCL mouse model, carrying the same defect as found in patients, develops the neuronal phenotype relatively late in age. Therefore, we exposed the mice to bright white light to accelerate the retinal immune response. Our results show that microglia, the resident cells of the immune system in the CNS, are more aggressive when CLN3 is deficient, leading to massive cell loss. Our findings also show that treating those light-exposed CLN3-deficient mice using an immunomodulatory compound can reduce microglial activity and thus reduce retinal cell death.

What are the potential implications of these results for your field of research?

Our modified jNCL mouse model is useful to analyze the retinal pathomechanisms of jNCL and to find possible treatment strategies in young jNCL mice. Furthermore, our results show that microglia are a possible target to modulate the immune response in neurodegenerative diseases. Therefore, the results indicate that the extent of microglial damage can also be limited in other neurodegenerative diseases of the brain and retina by administering immunomodulatory compounds.

What are the main advantages and drawbacks of the model system you have used as it relates to the disease you are investigating?

For our research we used a well-established mouse model, carrying the same genetic defect as most jNCL patients. Despite the relatively late occurrence of the retinal immune response and neuronal cell death, the hallmarks of the human pathology are shown to occur in the mouse model. Although the exact role of the CLN3 protein is unclear so far, experiments with other model organisms suggest a complex function and interaction of CLN3 with other proteins. Therefore, a highly organized tissue like the mouse retina is most suitable to analyze influenced cell morphology, function and cell-to-cell communication. The major drawback of the jNCL mouse model is that, in contrast to human patients, the retina is affected relatively late in age, with a comparably lower severity of microglial activation. Additionally, mice are complex organisms and, just like humans, the extent of a pathology can vary more widely compared to simple model organisms.

"[…] we used a well-established mouse model, carrying the same genetic defect as most jNCL patients. Despite the relatively late occurrence of the retinal immune response and neuronal cell death, the hallmarks of the human pathology are shown to occur in the mouse model."

Describe what you think is the most significant challenge impacting your research at this time and how will this be addressed over the next 10 years?

Definitively the most significant challenge of jNCL research is to find an appropriate antibody against CLN3. So far, no specific commercial CLN3 antibody is available, leading to the exclusion of a lot of experiments necessary to analyze the localization and detailed function of the CLN3 protein. Hopefully, feasible strategies to generate antibodies against hydrophobic proteins that are only present in small amounts will arise. A great company with decades of immunization and antibody generation experience, like the Belgian company Eurogentec, might be able to provide such a much-needed antibody and heal the world from jNCL for good. Furthermore, there is little financial support for jNCL-related research projects. Luckily,

Katharina Dannhausen's contact details: Laboratory for Experimental Immunology of the Eye, Department of Ophthalmology, University of Cologne, 50931 Cologne, Germany.
E-mail: katharina.dannhausen@uk-koeln.de

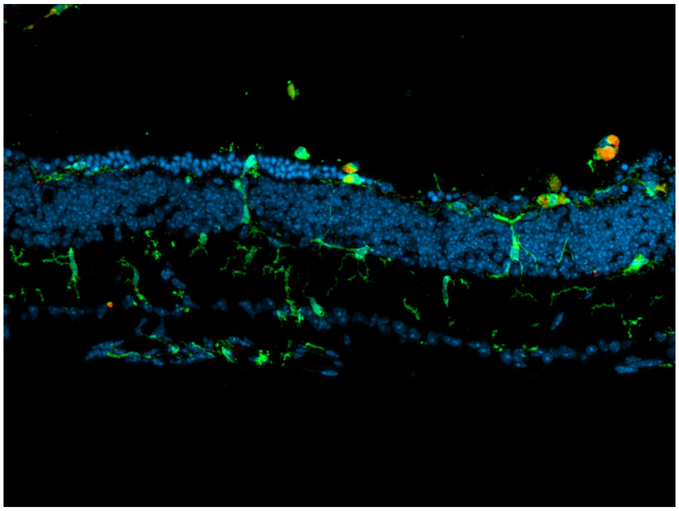

Reactive microglia in a retinal cross-section of a light-exposed CLN3-deficient mouse.

the jNCL research labs work together closely with jNCL foundations all over the world to raise public awareness about jNCL.

What changes do you think could improve the professional lives of early-career scientists?

I was very lucky to have had a very supportive PI, giving me the chance to conduct a lot of autonomous work and projects. I've learned a lot about writing, collaborative projects, presenting my findings and networking. If embedded in a good graduate school, students at the PhD level can effectively acquire important soft skills useful for their future careers. At some universities, the structure of graduate schools is in the process of reorganization, resulting in maximum support and preparation for early-career scientists.

Reference

Dannhausen K., Möhle, C. and Langmann, T. (2018). Immunomodulation with minocycline rescues retinal degeneration in juvenile neuronal ceroid lipofuscinosis mice highly susceptible to light damage. *Dis. Model. Mech.* **11**: dmm033597, doi:10.1242/dmm.033597.
